# Pre-transplant parathyroid hormone serum levels as predictors of kidney function after one year: a retrospective cohort study

**DOI:** 10.31744/einstein_journal/2025AO1392

**Published:** 2025-08-25

**Authors:** Lucas de Moraes Soler, Mariana Farina Valiatti, Mariana Moraes Contti, Hong Si Nga, Carolina da Costa Silva Porto, Júlio Cesar Garcia Alencar, Luís Gustavo Modelli de Andrade

**Affiliations:** 1 Universidade Estadual Paulista "Júlio de Mesquita Filho" Department of Internal Medicine Botucatu SP Brazil Department of Internal Medicine, Universidade Estadual Paulista "Júlio de Mesquita Filho", Botucatu, SP, Brazil.; 2 Universidade de São Paulo Faculdade de Medicina de Bauru Bauru SP Brazil Faculdade de Medicina de Bauru, Universidade de São Paulo, Bauru, SP, Brazil.

**Keywords:** Kidney transplantation, Glomerular filtration rate, Parathyroid hormone

## Abstract

**Objective:**

This study aimed to evaluate the association between pre-transplant parathyroid hormone levels and renal function 1-year after kidney transplantation.

**Methods:**

This retrospective cohort study was conducted at a tertiary public university hospital and included all patients who underwent kidney transplantation between May 2012 and September 2020. Patients under 18 years of age and those who received a transplant from a living donor were excluded. The primary outcome was renal function at 1 year, as assessed using the CKD-EPI formula. Univariate and multivariate analyses were performed to identify predictors of graft function at one year clinical and laboratory variables.

**Results:**

During the study period, 1,128 kidney transplants were performed. After applying exclusion criteria, 382 patients from six dialysis centers were included in the final analysis. Pre-transplant parathyroid hormone levels were not associated with renal function in either univariate or multivariate analyses (p=0.968 and p=0.616, respectively). In the multivariate analysis, recipient age (Coefficient: −0.41, 95% CI= −0.63 to −0.20, p<0.001) and Kidney Donor Profile Index (Coefficient: −0.44, 95%CI= −0.54 to −0.33, p<0.001) were significantly associated with graft function at 1 year.

**Conclusion:**

Pre-transplant serum levels of intact parathyroid hormone levels were not associated with renal function 1 year after transplantation. Conversely, recipient age and Kidney Donor Profile Index showed significant associations with graft outcomes, highlighting the importance of individualized risk assessment in transplant evaluation.

## In Brief

Pre-transplant parathyroid hormone levels were not associated with renal function 1 year after kidney transplantation. However, recipient age and the Kidney Donor Profile Index were independent predictors of graft function, highlighting the need for individualized risk assessment.

## Highlights

■ Pre-transplant parathyroid hormone levels were not linked to kidney function at 1 year post-transplant.
■ Recipient age showed a negative association with renal function at 12 months.
■ The Kidney Donor Profile Index was a strong predictor of graft function.
■ Individualized risk assessment remains essential for kidney transplant prognosis.

## Graphical abstract

**Figure:**
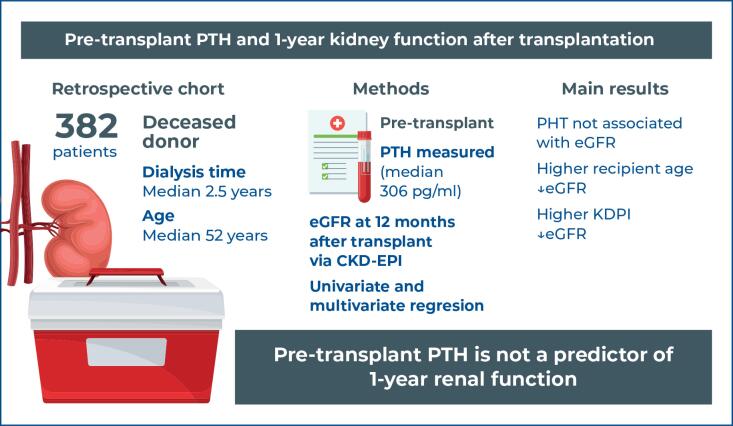

## INTRODUCTION

Renal replacement therapy (RRT) is the primary treatment for patients with end-stage chronic kidney disease (CKD), delivered through dialysis or kidney transplantation.^(1)^ Kidney transplantation is the preferred option for patients with end-stage CKD, as it can reverse various complications of kidney disease, reduce morbidity, and improve patient survival.^(2)^

As CKD progresses, its complications become increasingly prevalent and severe.^(3)^ One such complication is the bone mineral disease (BMD), a systemic disorder involving biochemical alterations (in calcium, phosphorus, parathyroid hormone [PTH], and vitamin D), abnormalities in bone turnover, and extraskeletal calcification.^(3,4)^ These biochemical disturbances can lead to secondary hyperparathyroidism (SHP), a condition with complex pathogenesis involving vitamin D deficiency, increased levels of fibroblast growth factor 23, hypocalcemia, and hyperphosphatemia. SHP can result in significant abnormalities in bone mineralization and turnover.^(4)^

In SHP, the progressive drop in serum vitamin D levels, associated with the loss of kidney function, causes PTH to increase in an attempt to maintain calcium and phosphorus homeostasis. As CKD advances, continuous stimulation of the parathyroid tissue can lead to hyperplasia and the formation of adenomas in the gland, as well as changes in the expression of calcium-sensitive vitamin D receptors. This disrupts PTH regulation, leading to persistently elevated hormone levels.^(5)^ Elevated PTH contributes to BMD in CKD and increases cardiovascular morbidity and mortality by accelerating atherosclerosis and vascular calcification.^(3)^

Kidney transplantation can reverse SHP.^(6)^ PTH levels typically drop by the end of the first year post-transplant, with the decrease being greater in the first 3 months. However, 20-50% of patients may still exhibit elevated PTH levels after 1 year, despite normalized kidney function, due to irreversible parathyroid hyperplasia. This condition is characterized by parathyroid gland hyperplasia of that no longer responds to feedback inhibition by vitamin D and calcium.^(7–9)^

Post-transplant BMD is attributed to high PTH levels acting on a now-functioning kidney.^(10)^ Parathyroid hormone induces hypercalcemia by increasing tubular and intestinal calcium reabsorption and promoting bone reabsorption. Simultaneously, it reduces phosphorus reabsorption in the proximal tubule, causing hypophosphatemia. Persistent elevation of PTH may contribute to graft loss, likely due to tubulointerstitial calcification and renal artery vasoconstriction.^(11–15)^

## OBJECTIVE

To evaluate the correlation between pre-transplant serum parathyroid hormone levels and renal graft function 1-year after transplantation.

## METHODS

### Study design, setting and participants

This retrospective cohort study was conducted at a tertiary care center and included all individuals who underwent kidney transplantation at *Hospital das Clínicas, Faculdade de Medicina de Botucatu* between May 2012 and September 2020. Patients were excluded if they received a kidney from a living-donor or were under 18 years of age.

The primary outcome was estimated glomerular filtration rate (eGFR), calculated using the CKD-EPI equation 1 year after transplantation. The eGFR is a routinely used clinical estimate of kidney function, based on serum creatinine and variables such as age and sex. It does not represent a direct measurement of the glomerular filtration rate (GFR).^(16)^

The study was approved by the Local Ethics Committee of the *Faculdade de Medicina de Botucatu* (CAAE: 05762818.8.0000.5411; # 3.164.339). Informed consent was waived due to the retrospective nature of the study. This manuscript adheres to the Reporting of Observational Studies in Epidemiology (STROBE) guidelines.^(17)^

### Variables and measurements

Demographic and transplant-related data were retrieved from the HCFMB Kidney Transplant Service data system (DataTx).

Receptor variables included race, sex, underlying disease, dialysis method, duration of RRT, single-pool Kt/V, calcium, phosphorus, hemoglobin, and serum intact PTH. Laboratory data corresponded to the most recent routine test available closest to the transplantation date, or within 6 months prior. kt/V, a measure of dialysis dose, was calculated using the Daugirdas equation.^(18)^

Transplant-related variables included panel, number of mismatches, donor cause of death and comorbidities, use of vasoactive drugs, induction drug type, initial immunosuppression, cold ischemia time, presence of delayed graft function, Kidney Donor Profile Index (KDPI),^(19–21)^ and creatinine 1 one year, which was used to calculate the CKD-EPI.

### Statistical analysis

Categorical variables are expressed as absolute numbers and percentages and analyzed using Pearson's χ^2^ test. Continuous variables were assessed for normality using the Shapiro-Wilk test. They were presented as medians and interquartile ranges (for non-normally distributed data) or as means and standard deviations (for normally distributed data), and analyzed using the Mann-Whitney or Student's *t*-test, respectively.

Univariate linear regression was conducted to assess the associations between pre-transplant and transplant-related variables and eGFR after 1 year. For patients who experienced graft loss or death, an eGFR of zero was assigned. PTH levels were included in the analysis on a natural logarithmic scale (logn).

Multivariate linear regression included variables from the univariate analysis (p <0.15) and pretransplant PTH levels (regardless of the p-value in the univariate analysis) to analyze their association with kidney function after 1 year.

Survival analysis, considering graft loss and death, was performed using Kaplan-Meier curves based on median pre-transplant PTH levels.

All statistical analyses were conducted using R software, version 3.3.2, with p<0.05 considered statistically significant.

## RESULTS

### Participants

**Figure 1:**
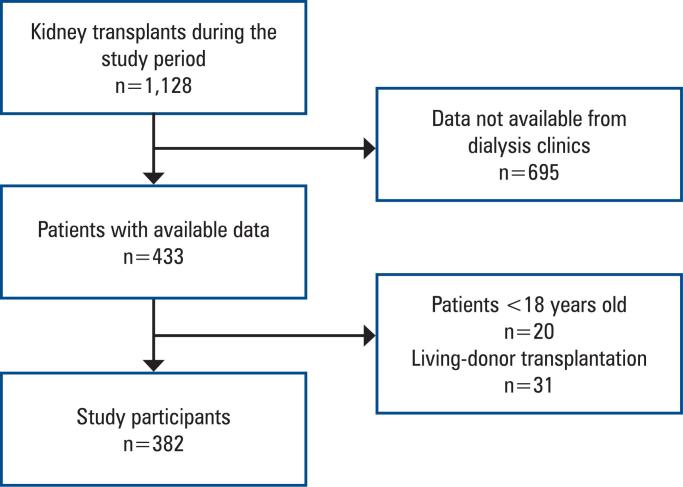
Patient flowchart of the study

During the study period, a total of 1,128 kidney transplants were performed at HCFMB. Pre-transplant data were collected from six dialysis centers, ensuring a diverse representation of patients from various clinical backgrounds. After applying the exclusion criteria, 51 patients were excluded from the final analysis due to incomplete data or loss to follow-up (Figure 1). Consequently, 382 patients were included in this study, forming a well-defined cohort for statistical analysis (Table 1).

**Table 1:** Baseline characteristics of kidney transplant recipients

Patient characteristics		
Receiver characteristics		
	Age, median (IQR), years	52 (40-60)
	Female, n (%)	166 (43)
Race, n (%)		
	White	265 (69)
	Black or African American	48 (13)
	Multiple	67 (18)
	Asian	2 (0.5)
	Time on dialysis, median (IQR), months	33 (20-54)
Underlying disease, n (%)		
	Diabetes nephropathy	93 (24)
	Hypertension	83 (22)
	Glomerulonephritis	69 (18)
	Undetermined	86 (23)
	Other (urological causes, polycystic kidney disease, etc.)	51 (13.3)
Dialysis method, n (%)		
	Hemodialysis	357 (93.5)
	Peritoneal	25 (6.5)
	Pre-transplant PTH, median (IQR)	306 (169-564)
	Pre-transplant calcium, median (IQR)	8.80 (8.30-9.38)
	Pre-transplant phosphorus, median (IQR)	4.90 (4.20-6.07)
	Hemoglobin pre-transplant, median (IQR)	11.40 (10.40-12.40)
	Kt/V pre-transplant, median (IQR)	1.27 (1.10-1.52)
	Panel, median (IQR)	0 (0-23)
Donor characteristics		
	Age, median (IQR), years	44 (32-51)
Cause death, n (%)		
	Cerebrovascular disease	198 (52)
	Traumatic brain injury	151 (40)
	Others	33 (8.6)
Underlying disease, n (%)		
	Hypertension	121 (32)
	*Diabetes mellitus*	15 (3.9)
	Use of vasoactive drugs, n (%)	240 (63)
	Creatinine, median (IQR)	1.20 (0.88-1.70)
	KDPI, median (IQR)	46 (25-70)
Transplant-related characteristics of the recipient		
	Mismatches, median (IQR)	3 (2-3)
Induction medication, n (%)		
	Basiliximab	114 (30)
	Thymoglobulin	261 (70)
Immunosuppressive drugs, n (%)		
	Tacrolimus	363 (95)
	Mycophenolate	211 (55)
	Azathioprine	11 (2-9)
	mTOR inhibitors 121 (32%)	
	Cold ischemia time, median (IQR)	22 (20-24)
DGF, n (%)		
	No	143 (37)
	Yes	221 (58)
	Days of hospitalization (IQR)	14 (10-21)
Outcomes		
	Glomerular filtration rate at 12 months, median (IQR)	54 (34-74)
	Death in 12 months, n (%)	98 (26)

IQR, interquartile range; KDPI: Kidney Donor Profile Index; DGF: Delayed graft function.

The median age of the recipients was 52 years (IQR: 40-60) with 57% being male. The most prevalent underlying causes of end-stage kidney disease (ESKD) among the included patients were diabetic nephropathy (24%), glomerulonephritis (18%), and hypertensive nephropathy (22%). The median duration of dialysis before transplantation was 33 months (IQR: 20-54), with 93.5% of patients undergoing hemodialysis and 6.5% receiving peritoneal dialysis.

Regarding pre-transplant biochemical parameters, the median PTH level was 306 pg/mL (IQR: 169-564). Median serum calcium and phosphorus levels were 8.8 mg/dL (IQR: 8.30-9.38) and 4.90 mg/dL (IQR: 4.20-6.07), respectively. The median hemoglobin level was 11.40 g/dL (IQR: 10.40-12.40), reflecting the frequent presence of anemia in this population.

### Association between pre-transplant PTH and kidney function at 1 year

Pre-transplant PTH levels were not significantly associated with kidney function at 1 year, as shown in both univariate (p=0.968) and multivariate (p=0.616) linear regression analyses (Table 2).

**Table 2:** Univariate and multivariate analysis of predictors of kidney function at one year post-transplant

Variable		Univariate analysis (95%CI)	p value	Multivariate analysis (95%CI)	p value
Recipient age (years)		−0.43 (−0.66 to −0.19)	<0.001	−0.41 (−0.63 to −0.20)	<0.001
Sex					
	Male	5.70 (−0.41 to 11.81)	0.068	6.36 (0.78 to 11.94)	0.026
Race/Ethnicity					
	White	Reference Group		Reference Group	
	Black or African American	2.12 (−7.21 to 11.46)	0.655	Not included	
	Multiracial	4.83 (−3.30 to 12.97)	0.243	Not included	
	Asian	9.62 (−32.61 to 51.85)	0.654	Not included	
Time on dialysis (years)		−0.07 (−0.15 to 0.00)	0.065	−0.04 (−0.11 to 0.03)	0.292
Underlying Disease					
	*Diabetes mellitus*	Reference Group		Reference Group	
	Glomerulonephritis	−3.60 (−13.03 to 5.84)	0.454	Not included	
	Hypertension	−3.84 (−12.81 to 5.12)	0.400	Not included	
	Undetermined	3.79 (−5.09 to 12.67)	0.402	Not included	
Panel reactive antibodies (%)		−0.06 (−0.16 to 0.04)	0.211	Not included	
HLA mismatches		−3.82 (−6.76 to −0.88)	0.011	−3.28 (−5.95 to −0.62)	0.016
Dialysis method before transplant					
	Hemodialysis	Reference Group		Reference Group	
	Peritoneal dialysis	11.94 (−0.31 to 24.18)	0.056	8.56 (−2.59 to 19.71)	0.132
Pre-transplant PTH (log-transformed)		−0.06 (−3.03 to 2.91)	0.968	−0.69 (−3.38 to 2.00)	0.616
Pre-transplant calcium (mg/dL)		2.40 (−1.03 to 5.84)	0.170	Not included	
Pre-transplant phosphorus (mg/dL)		0.92 (−1.06 to 2.90)	0.361	Not included	
Pre-transplant hemoglobin (g/dL)		−1.19 (−3.06 to 0.68)	0.212	Not included	
Pre-transplant Kt/V		4.54 (−3.34 to 12.42)	0.258	Not included	
Donor age (years)		−0.80 (−1.02 to −0.58)	<0.001	Not included	
Cause of donor death				Not included	
	Cardiovascular disease	Reference Group		Reference Group	
	Other causes	13.92 (3.19 to 24.64)	0.011	Not included	
	Traumatic brain injury	17.95 (11.79 to 24.12)	<0.001	Not included	
Donor hypertension		−13.43 (−19.83 to −7.03)	<0.001	Not included	
Donor *diabetes mellitus*		−26.28 (−41.72 to −10.84)	0.001	Not included	
Donor use of vasoactive drugs		−1.18 (−7.48 to 5.11)	0.712	Not included	
Final donor creatinine (mg/dL)		−1.34 (−4.35 to 1.66)	0.380	Not included	
Kidney Donor Profile Index		−0.46 (−0.57 to −0.35)	<0.001	−0.44 (−0.54 to −0.33)	<0.001
Induction therapy with thymoglobulin		−2.37 (−9.05 to 4.31)	0.486	Not included	
Cold Ischemia time (hours)		−0.30 (−1.04 to 0.44)	0.421	Not included	

GN: glomerulonephritis; PTH: parathyroid hormone; CVD: cardiovascular disease; TBI: traumatic brain injury; KDPI: Kidney Donor Profile Index; HLA: human leukocyte antigens.

In the multivariate model, the following variables were independently associated with lower eGFR at 1 year: recipient age (coefficient: −0.41; 95%CI= −0.63 to −0.20; p<0.001), number of human leukocyte antigens mismatches (coefficient: −3.28; 95%CI= −5.95 to −0.62; p=0.016), and KDPI (coefficient: −0.44; 95%CI= −0.54 to −0.33; p<0.001).

Other pre-transplant biochemical parameters, including serum calcium (p=0.170), phosphorus (p=0.361), hemoglobin (p=0.212), and Kt/V (p=0.258), were not significantly associated with kidney function at 1 year.

### Survival analysis

**Figure 2:**
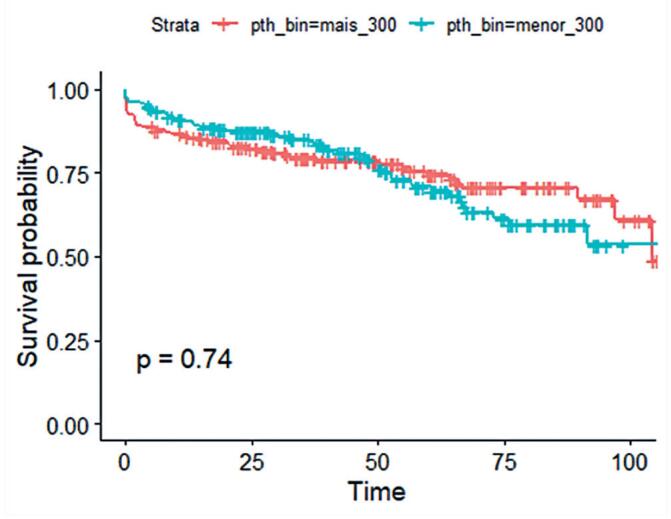
Kaplan-Meier survival curves showing the probability of survival over time (years) for groups stratified by median pre-transplant parathyroid hormone levels

A Kaplan-Meier survival analysis was performed to evaluate graft and patient survival over the follow-up period. Patients were stratified according to the median pre-transplant PTH level (300 pg/mL), and survival probabilities were compared between groups (Figure 2). The log-rank test showed no significant difference in graft survival between the groups (p=0.74).

These findings suggest that, although persistent hyperparathyroidism after transplantation has been associated with poor graft outcomes, pre-transplant PTH levels alone do not appear to be a major determinant of graft longevity. Future studies should explore the longitudinal trajectory of post-transplant PTH levels and their impact on renal function.

The log-rank test was used to assess statistical significance, with p<0.05 considered statistically significant.

## DISCUSSION

This retrospective cohort study, which included 382 patients who underwent kidney transplantation at HCFMB between 2012 and 2020, found no association between pre-transplant PTH levels and renal function at 1 year. Although previous studies have suggested a potential link between elevated PTH levels and adverse post-transplant outcomes, our findings challenge this hypothesis. The lack of a statistically significant correlation highlights important considerations regarding the role of mineral metabolism disturbances in kidney transplantation and suggests that pre-transplant PTH alone may not be a reliable predictor of graft function.

Previous studies have indicated that high pre-transplant PTH levels may contribute to graft loss and return to dialysis, possibly through mechanisms such as vascular calcification, tubulointerstitial fibrosis, and endothelial dysfunction.^(22)^ Chronic imbalances in mineral metabolism during dialysis may cause irreversible damage that affects graft survival. However, our study differs from previous investigations in several important ways. First, we analyzed PTH levels as a continuous variable rather than using arbitrary cutoffs, ensuring a more precise evaluation of their impact on renal function. Second, we used the CKD-EPI formula, a validated method for estimating GFR, which may than equations used in earlier studies.^(23)^

Despite the biological plausibility of an association between elevated pre-transplant PTH levels and graft dysfunction—via mechanisms such as hypercalcemia-induced vasoconstriction, tubulointerstitial calcification, and phosphate imbalance—our results did not support this link.^(24,25)^ One possible explanation is that post-transplant management, including the use of vitamin D analogs, phosphate binders, and cinacalcet, may mitigate the potential adverse effects of preexisting hyperparathyroidism, resulting in improved metabolic control and reduced complications.^(26)^

Interestingly, while our study did not identify a relationship between pre-transplant PTH levels and graft function, persistently elevated post-transplant PTH levels have been associated with poorer renal outcomes.^(27)^ This distinction is clinically relevant, as it emphasizes the need to monitor PTH levels beyond the pre-transplant period, particularly in patients with persistent hyperparathyroidism (pHPT). Prior studies have identified several risk factors for pHPT, including prolonged dialysis duration, disrupted calcium-phosphate homeostasis, and previous cinacalcet use.^(23)^ However, in our multivariate analysis, dialysis duration was not associated with 1-year renal function, nor were serum phosphorus, calcium, or hemoglobin levels. These findings suggest that although metabolic disturbances are common among transplant candidates, their impact on short-term post-transplant outcomes may be less pronounced than previously believed.

Consistent with previous reports, our findings contribute to the ongoing debate regarding the role of hyperparathyroidism in kidney transplantation. Evenepoel et al. highlighted the lack of consensus on managing hyperparathyroidism in transplant candidates, reporting that American dialysis centers hold divergent views on whether elevated pre-transplant PTH levels negatively affect graft survival.^(26)^ While many centers consider severe hyperparathyroidism a risk factor for post-transplant complications, others prioritize kidney allocation without specifically factoring PTH levels. Given this uncertainty, our study adds new insights by suggesting that pre-transplant PTH levels alone may not be a decisive factor in transplant outcomes. Future research should focus on the long-term trajectory of post-transplant PTH levels, as persistent hyperparathyroidism has been associated with increased risks of cardiovascular complications, fractures, and bone mineral disorders— all of which may indirectly affect graft survival.^(28)^

Although PTH was not a predictor of graft function in our cohort, several recipient- and donor-related factors showed significantly influenced renal outcomes at 1 year. In particular, our multivariate analysis identified that the Kidney Donor Profile Index (KDPI) was strongly associated with graft function, reinforcing its role as a validated predictor of transplant outcomes in multiple settings, including Brazilian cohorts.^(20)^ The KDPI integrates multiple donor factors, such as age, creatinine levels, and cause of death, providing a comprehensive risk assessment. In addition, recipient age emerged as a significant factor influencing kidney function, consistent with previous studies reporting lower eGFR values in older recipients.^(20)^ This association is likely multifactorial, involving age-related changes in immune response, increased comorbidities, and differences in organ allocation strategies. However, it remains unclear whether younger recipients inherently achieve better renal outcomes, or this advantage is primarily driven by the allocation of higher-quality kidneys to younger patients.^(20)^

Given the complexity of the factors influencing graft function, our findings highlight the need for individualized risk assessment in kidney transplant recipients. Future studies should explore the long-term effects of persistent hyperparathyroidism on renal function and overall survival, whether pre-transplant interventions such as parathyroidectomy or cinacalcet use can improve post-transplant outcomes, and how metabolic disturbances interact with immune-mediated factors to influence long-term graft survival. Further studies exploring the quantitative relationships between donor, recipient, and surgical procedure variables and renal function are essential to deepen our understanding of their prognostic implications and to guide clinical decision-making.

### Limitations

Despite its strengths, this study has several limitations. First, its retrospective design may introduce selection bias and limits the ability to establish causality. Second, post-transplant PTH levels were not assessed, preventing direct comparison between pre- and post-transplant dysregulation of mineral metabolism. Third, this was a single-center study, which may limit the generalizability of the findings to broader transplant populations.

Nevertheless, our study offers valuable insights into the role of pre-transplant PTH in kidney transplantation, suggesting that recipient age, human leukocyte antigens mismatch, and KDPI play more significant roles in early graft function than pre-transplant mineral metabolism parameters.

## CONCLUSION

Pre-transplant serum parathyroid hormone levels were not associated with graft function at 1 year post-kidney transplantation. However, recipient age and kidney donor profile index showed significant associations with graft function. These findings suggest that while parathyroid hormone alone may not serve a reliable predictor of post-transplant outcomes, individualized risk assessment incorporating both recipient and donor characteristics remains essential in evaluating transplant prognosis.
